# Experience of applying cosmetic Etafilcon A contact lens in cases with microcornea

**DOI:** 10.5935/0004-2749.20210075

**Published:** 2021

**Authors:** Ferah Ozcelik, Cigdem Altan, Asli Kirmaci Kabakci, Semih Urvasizoglu

**Affiliations:** 1 University of Health Science Beyoglu Eye Training and Research Hospital, Istanbul, Turkey; 2 University of Health Science Okmeydani Training and Research Hospital, Istanbul, Turkey; 3 Eyup State Hospital, Istanbul, Turkey

**Keywords:** Microcornea, Contact lens, hydrophilic, Cosmetic techniques, Microcórnea, Lentes de contato hidrofílicas, Técnicas cosméticas

## Abstract

**Purpose:**

Microcornea is a rare condition that frequently resulets in serious cosmetic concerns due to the resultant asymmetrical appearance of the eye, and its cosmetic rehabilitation is possible with the use of colored contact lenses. This paper aims to present our experiences with the use of cosmetic Etafilcon A contact lenses for microcornea.

**Methods:**

Eight patients with unilateral microcornea without any systemic involvement were included in this study, and they underwent routine ophthalmological examination, corneal topography, and optical biometry. We applied the cosmetic Etafilcon A contact lens (1-DAY ACUVUE^®^ DEFINE^®^ with Lacreon^®^) of the same edge color to the patients. The levels of satisfaction in terms of cosmesis and comfort were evaluated with the use of visual analog scales (VAS).

**Results:**

In the patients, the corneal diameter asymmetry was acceptably adjusted, and each of the patients reported extreme satisfaction. The mean VAS score was 8.9 ± 1.0 (range: 7-10) for the cosmetic satisfaction rate and 8.4 ± 1.0 (range: 7-10) for the comfort rate. The patients obtained the best-corrected visual acuity without or with additional eye-glasses. None of the patients complained about vision issues under photopic and scotopic conditions.

**Conclusion:**

1-DAY ACUVUE^®^ DEFINE^®^ with Lacreon^®^ lens has promising satisfactory cosmetic outcomes along with visual enhancement in cases of microcornea. This is the first study to report the use of this lens for the cosmetic rehabilitation of patients with microcornea.

## Abstract (pt)

**Objetivo:**

A microcórnea é uma afecção rara, que frequentemente causa graves queixas estéticas devido a uma aparência assimétrica, mas passível de reabilitação estética através de lentes de contato coloridas. O objetivo deste estudo é apresentar nossas experiências no uso de lentes de contato cosméticas Etafilcon A em casos de microcórnea.

**Métodos:**

Oito pacientes com microcórnea unilateral, sem acometimento sistêmico, foram incluídos e submetidos a exame oftalmológico de rotina, topografia corneana e biometria óptica. Aplicamos nos pacientes uma lente de contato cosmética Etafilcon A (1-Day Acuvue^®^ Define^®^ com Lacreon^®^) da mesma cor da borda da córnea dos pacientes. Os níveis de satisfação em termos de estética e conforto foram avaliados por meio de escalas visuais analógicas (EVA).

**Resultados:**

A assimetria do diâmetro da córnea foi corrigida em um grau aceitável e todos os pacientes ficaram muito satisfeitos. A pontuação média da EVA foi de 8,9 ± 1,0 (variação: 7-10) para o grau de satisfação estética e 8,4 ± 1,0 (intervalo: 7-10) para o grau de conforto. Os pacientes obtiveram a melhor acuidade visual corrigida com ou sem óculos adicionais. Não houve queixas visuais em condições fotópicas ou escotópicas.

**Conclusão:**

A lente 1-Day Acuvue^®^ Define^®^ com Lacreon^®^ é promissora em termos de resultados estéticos satisfatórios, bem como de melhoria da visão em casos de microcórnea. Este é o primeiro estudo a relatar o uso dessa lente na reabilitação cosmética de pacientes com microcórnea.

## INTRODUCTION

Microcornea is an abnormality of the corneal size where the corneal diameter is <11 mm on the horizontal meridian. This disorder may be unilateral or bilateral, and it may follow an autosomal dominant or an autosomal recessive pattern. The probable mechanism underlying microcornea is that it occurs secondary to an arrest in the corneal development due to overgrowth of the tips of the optic cup. Usually, cornea is clear, the corneal curvature is flatter than that in the normal eyes, and the patients tend to be hyperopic. Significant asymmetry in the corneal diameter of the same patient is considered abnormal, even when both the corneal diameters fall within the normal range^(1-3)^.

Isolated microcornea is an extremely rare condition that presents without any other significant ocular or systemic findings^(2,3)^. Infantile glaucoma and angle-closure or open-angle glaucoma may co-occur with microcornea. Cataract may be encountered at the birth or may develop later in the life. The other associated ocular conditions include amblyopia, iris colobomas, corectopia, microphacia, persistent fetal vasculature, rod-cone dystrophy, retinopathy of prematurity, myopic chorioretinal atrophy, posterior staphyloma, and optic nerve hypoplasia^(4-6)^.

The systemic associations in microcornea include Rieger syndrome, Nance-Horan syndrome, Marfan syndrome, Ehlers-Danlos syndrome, Weill-Marchesani syndrome, trisomy 21, Turner syndrome, Norrie syndrome, Warburg syndrome, cataract-microcornea syndrome, and renal glucosuria^(7-9)^.

In addition to all these findings and syndromes, microcornea is a major cause of cosmetic concern. Fortunately, the rehabilitation of this condition is possible with the use of cosmetic contact lenses.

1-DAY ACUVUE DEFINE with Lacreon contact lens (Johnson & Johnson Vision Care) is a variant of the 1-DAY ACUVUE family of etafilcon A hydrogel lenses, with an additional enclosed peripheral limbal ring of pigments beneath the front lens surface in the lens matrix^(10-12)^. This limbal ring is about 5-mm wide, extending as a concentric ring of 7-12-mm diameter in the lens periphery, with the dimensions of the ring differing slightly based on the variant type^(13)^. This contact lens (CL) is available in 2 different designs: Natural Shimmer that has a brown color and Natural Sparkle that has a blue color. The power range of the lens is -0.25 to -6.00 D (0.25 steps) and -6.50 to -9.00 D (0.50 steps) in the myopic range and +0.50 and +1.00 D in the hyperopic range. The base curve for this CL is 8.5 mm, and the diameter is 14.2 mm.

The purpose of this study is to present our experiences of using the cosmetic Etafilcon A contact lenses for correcting cases of microcornea.

## METHODS

Eight patients with unilateral microcornea who were applied with the 1-DAY ACUVUE® DEFINE® with Lacreon^®^ contact lens at our contact lens department were included in this study. Informed consent was obtained from each subject before their enrollment. The protocol of the study was approved by the local ethical committee and it also adhered to the ethical principles stated in the ‘Declaration of Helsinki’.

The characteristics of the patients such as age, gender, laterality, and the history of ocular and systemic diseases were recorded. Routine ophthalmological examination including the uncorrected visual acuity (UCVA) and the best-corrected visual acuity (BCVA) testing before the CL fitting, refractive error measurement, slit-lamp biomicroscopy, IOP measurement using Goldmann applanation tonometry, and fundus examination with +90 D lens was performed for each participant before their corneal topographic analysis. The keratometry values and the white-to-white corneal diameter readings by Sirius corneal topography (SCHWIND eye-tech-solutions GmbH, Kleinostheim), as well as the measurements of axial length (AL) and anterior chamber depth (ACD) by the AL-Scan optical biometer (NIDEK Co.; Gamagori, Japan) were recorded for each patient.

**Figure 1:**
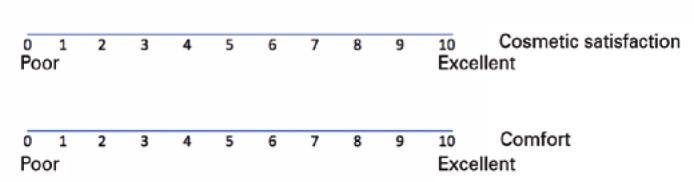
Visual analog scales employed to record the subjective perceptions of the study participants regarding their cosmetic satisfaction and comfort.

The 1-DAY ACUVUE^®^ DEFINE^®^ with Lacreon contact lens with brown edge color (Shimmer) was applied to each patient. The procedure for contact lens fitting was conducted as instructed by the technical fitting guide and the manufacturer’s specifications. The other aspects of the contact lens fitting including movement and push-up test were also assessed to ensure an acceptable fit. The subjects with inappropriate contact lens fits were considered ineligible for this study. The base curve of the lens was 8.5 mm and the diameter was 14.2 mm in all cases, considering that it was the only available option for 1-DAY ACUVUE^®^ DEFINE^®^ with Lacreon contact lens. The data related to the contact lens powers, visual acuities obtained with the CL, and the use of additional eye-glasses, when required, were recorded. No contact lens care solutions or any other contact lens care products were used in this study. At the 1-week, 4-week, and 12-week follow-up visits, the patients’ satisfaction and persistence statuses were queried, and the development of any complications was assessed. The visual analog scales (VAS) were administered to the patients 1 week after their first visit in order to record their subjective cosmetic satisfaction and comfort rates on a scale of 0 (poor) to 10 (excellent). The scale measured exactly 10 cm, was horizontally oriented, and the values for statistical analyses were measured at the point where the mark inserted by the patient crossed the scale. Figure 1 depicts an example of the scale used in this study. The VAS has been commonly used to assess contact lens adaptation ^(14,15)^.

## RESULTS

All 8 patients who visited our polyclinic with cosmetic complaints because of their small eyes since birth were included in this study. None of the patients had any history of systemic diseases nor a family history of microcornea. Only 1 patient (Case 4) had a history of congenital cataract surgery, while the others had isolated microcornea. Two of the patients developed strabismus in their primary gaze (Cases 4 and 8). In the patients’ healthy eyes without microcornea, all examination findings were unremarkable and the visual acuities were 1.0. All pupillary examinations were normal. All the patients had brown iris color. The intraocular pressure measurements by Goldmann tonometry fell within the normal range without the use of any medications. Biomicroscopic, retinal, and optic disc examinations were unremarkable, except for the unilateral small corneas (≤10.8 mm horizontal diameter). None of the patients had a history of using cosmetic contact lens.

The assessment of contact lens fitting by slit-lamp revealed an acceptable fit for all patients in terms of its centration, movement, and tightness. All 8 patients reported amblyopia in their eyes with microcornea. Two of the patients were prescribed with eye-glasses in addition to the CL due to the lack of proper CL power. The BCVA was achieved in all of the patients fitted with the CL, without or with the addition of eye-glasses. The BCVA visual acuities without or with additional eye-glasses (if applicable) and the other features are shown in table 1.

**Table 1:** Demographic characteristics and the examination results for the study patients

	Case 1	Case 2	Case 3	Case 4	Case 5	Case 6	Case 7	Case 8
Age (years)	19	16	42	20	25	39	23	15
Gender	Female	Female	Female	Female	Female	Female	Female	Female
Laterality	Left	Right	Left	Left	Right	Left	Right	Left
UCVA (Snellen)	0.15	0.1	0.15	HM	0.1	0.05	0.05	0.016
BCVA (Snellen)	0.5	0.6	0.6	HM	0.5	0.4	0.5	0.1
Refractive error	+4.00 (-0.25 115)	-3.00	-2.00 (-4.50 155)	+4.50	-2.75	-4.00	-3.50 (-0.25 136)	-8.50 (-0.50 18)
Contact lens power (D)	+1.00	-3.00	-2.00	plano	-2.75	-4.00	-3.50	-8.00
VA with CL (Snellen)	0.3	0.6	0.4	HM	0.5	0.4	0.5	0.1
VA with spectacle added to CL (Snellen)	0.5	none	0.6	none	none	none	none	none
VAS score (Cosmetic satisfaction-Comfort)	10-8	9-9	8-7	7-8	9-10	9-9	10-9	9-7
WtoW (mm)	10.54	10.42	10.75	10.50	10.81	10.54	10.47	10.84
K1 (D)	40.78	42.28	42.35	42.45	44.21	42.13	42.17	45.24
K2 (D)	41.32	42.81	46.13	43.00	44.45	42.92	43.01	47.97
Kavg (D)	41.05	42.54	44.24	42.72	44.33	42.52	42.59	46.56
AL (mm)	22.37	23.08	23.42	22.12	22.85	23.28	22.79	23.23
ACD (mm)	2.61	2.70	2.76	2.52	2.34	2.86	2.62	2.26
Additional ocular pathology	None	None	None	Past congenital cataract surgery, strabismus	None	None	None	Strabismus

(UCVA= Uncorrected Visual Acuity; BCVA= Best-Corrected Visual Acuity; D= Diopter; HM= Hand Motion; VA= Visual Acuity; CL= Contact Lens; VAS= Visual Analog Scale; WtoW= White-to-White corneal diameter; AL= Axial length; ACD= Anterior chamber depth).

**Figure 2:**
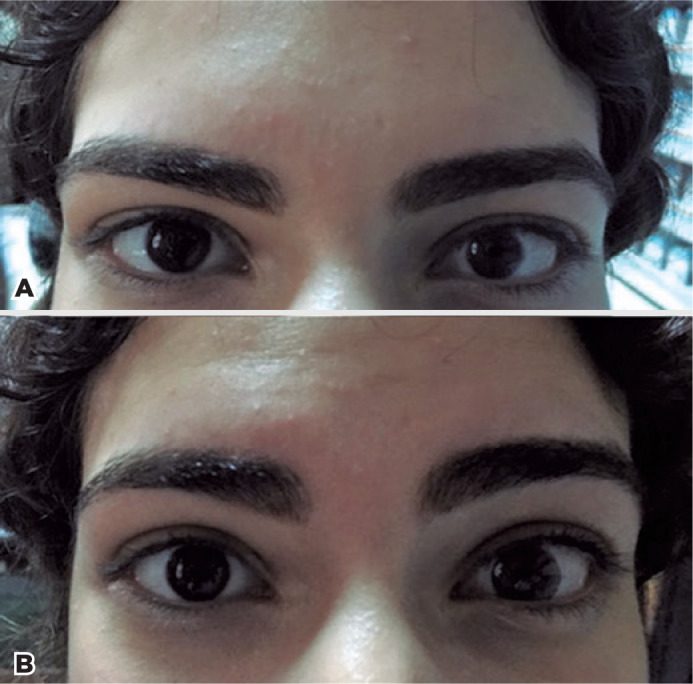
A) Patient with left microcornea (Case 1) before any contact lens fitting. B) Image of the same patient after the application of the contact lens.

**Figure 3:**
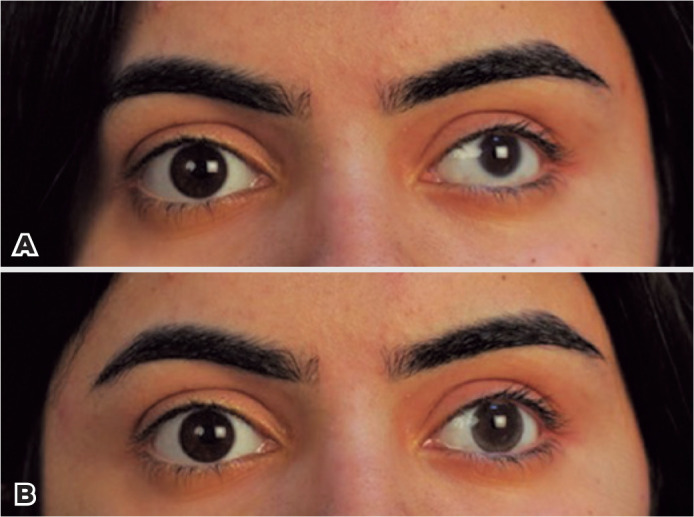
A) Patient with left microcornea (Case 4), before any contact lens fitting. B) Image of the same patient after the application of the contact lens.

1-DAY ACUVUE DEFINE with Lacreon contact lens provided comfortable wearing experience and improved cosmesis in addition to improved visual acuity in the majority of the patients by means of refractive error correction. The corneal diameter asymmetries were masked at acceptable levels (Figures 2A, 2B and 3A, 3B). Both the palpebral aperture and the marginal reflex were slightly increased after the CL adaptation, although not significantly. All the patients reported cosmetic satisfaction. They had no complaint regarding vision under the photopic and scotopic conditions. The mean VAS score was 8.9 ± 1.0 (range: 7-10) for the cosmetic satisfaction rate and 8.4 ± 1.0 (range: 7-10) for the comfort rate. No complications developed in any of the patients during the follow-up period. Except for 1 patient who could not sustain the use of the CL after the follow-up period due to economic considerations, all other patients preferred the continued use of the prescribed CL until the 1-DAY ACUVUE^®^ DEFINE^®^ with Lacreon contact lens was taken off the market from Turkey in the year 2017, in accordance with the marketing strategies of the brand.

## DISCUSSION

The 1-DAY ACUVUE DEFINE brand contact lens (Johnson & Johnson Vision Care) provides an additional enclosed peripheral limbal ring of pigments beneath the front lens surface in the lens matrix^(11-12)^. This limbal ring is approximately 5-mm wide, extending as a concentric ring of 7-12-mm diameter in the lens periphery^(13)^. The purpose of adding this limbal ring was to enhance the cosmetic appearance of the iris, to define a clear edge for the iris at the limbus, and to make the eye look naturally bigger^(13)^. Recently, a version with the added polyvinyl pyrrolidone (PVP) wetting agent, 1-DAY ACUVUE DEFINE with Lacreon, was launched globally. The LACREON^®^ Technology permanently embeds a water-holding ingredient, similar to that found in natural tears, into the proven etafilicon A material. The common use of this lens is to clarify the natural iris color. Our study is the first to report the use of this lens for the cosmetic rehabilitation of patients with microcornea.

Although the associated ocular abnormalities may affect the visual outcome; the satisfactory visual acuity levels can usually be reached in isolated microcornea patients via optical correction of the refractive errors and the treatment of amblyopia, if necessary. However, cosmetic concerns frequently constituted the significant portion of these patients’ complaints. We could achieve satisfactory cosmetic rehabilitation in all 8 patients with microcornea by fitting them with the 1-DAY ACUVUE DEFINE with Lacreon contact lenses. There are several other types of contact lenses being used for cosmetic problems, as reported in the literature, for example, ptosis by Katsoulos et al.^(16)^. To the best of our knowledge, the present study is the only example of the use of the 1-DAY ACUVUE DEFINE with Lacreon contact lenses for cosmetic rehabilitation. This contact lens enhances the natural beauty of the eye by providing a natural-looking definition to the limbal ring. We obtained satisfactory outcomes in terms of cosmetic improvement as well as visual enhancement in majority of our cases. In addition, by applying the concentric ring extending 7-12-mm in diameter in the lens periphery, the patients did not present with any visual issues under neither photopic nor scotopic conditions. However, two of the patients required eye-glasses in addition to their contact lens due to the lack of availability of the appropriate CL power (one of them had a refractive error >+1.00 D, and the other had astigmatism).

The level of visual improvement achieved was unfortunately limited for some of our patients due to the restricted range of power of the CL used. A higher level of visual acuity can be reached in such cases with the use of colored contact lenses with the appropriate power. For all of our patients, however, the visual acuities in the healthy eyes without microcornea were 1.0 without any refractive correction. This was the reason for their reluctance to use contact lenses for both the eyes, which would be the case with other types of colored contact lenses owing to the disadvantage of changing the natural eye color when compared to that with the 1-DAY ACUVUE^®^ DEFINE^®^ with Lacreon contact lenses. Our patients opted for better cosmetic results by using the 1-DAY ACUVUE^®^ DEFINE^®^ with Lacreon contact lens as it gives a natural look. Moreover, these two patients who used the eye-glasses were content with their asymmetry hiding effect. In patients requiring refractive error correction for both the eyes, colored contact lenses might be preferred.

Some studies in the past investigated the safety of this contact lens. For instance, Moezzi AM compared the out comes with the use of 1-DAY ACUVUE DEFINE, 1-DAY ACUVUE DEFINE with Lacreon, no lens wear, and a control lens with no tint; the authors found that the addition of PVP or pigments to etafilcon A to obtain a limbal ring design did not affect corneal swelling or limbal/bulbar hyperemia during the normal open-eye wear^(10)^. Galas et al. also demonstrated that the pigment colorant and the PVP embedded in the contact lens during autoclaving had no influence on the oxygen permeability of the etafilcon A material^(17)^. The authors reported that the printed portion of cosmetic contact lens is typically located in the periphery (or mid-periphery) of the lens; this region does not allow accurate measurement of Dk owing to the wide variations in the thickness values across a given lens’ profile^(14)^. We did not encounter any such complications in our patients during their follow-up examinations.

In conclusion, 1-DAY ACUVUE DEFINE with Lacreon contact lens can provide satisfactory levels of cosmetic rehabilitation in addition to enhancing the visual acuity in patients with microcornea. We suggest longer follow-up studies with a larger sample size to ascertain the benefits of this lens.
